# Multiple life-stage inbreeding depression impacts demography and extinction risk in an extinct-in-the-wild species

**DOI:** 10.1038/s41598-020-79979-4

**Published:** 2021-01-12

**Authors:** A. E. Trask, G. M. Ferrie, J. Wang, S. Newland, S. Canessa, A. Moehrenschlager, M. Laut, L. Barnhart Duenas, J. G. Ewen

**Affiliations:** 1 Institute of Zoology, Zoological Society of London, Regents Park, London, NW1 4RY UK; 2 Disney’s Animal Kingdom, Animals, Science and Environment, Lake Buena Vista, FL 32830 USA; 3 Sedgwick County Zoo, Zoo Boulevard, Wichita, KS 67212 USA; 4 Wildlife Health Ghent, Department of Pathology, Bacteriology and Avian Diseases, Faculty of Veterinary Medicine, Ghent University, Merelbeke, Belgium; 5 Centre for Conservation Research, Calgary Zoological Society, Zoo Road NE, Calgary, AB 2TE 7V6 Canada; 6 U.S. Fish and Wildlife Service, Pacific Islands Office, Honolulu, HI USA; 7 Division of Aquatic and Wildlife Resources, Guam Department of Agriculture, Mangilao, Guam

**Keywords:** Conservation biology, Ecological genetics, Population dynamics, Ecology, Genetics

## Abstract

Inbreeding can depress individuals’ fitness traits and reduce population viability. However, studies that directly translate inbreeding depression on fitness traits into consequences for population viability, and further, into consequences for management choices, are lacking. Here, we estimated impacts of inbreeding depression (*B*, lethal equivalents) across life-history stages for an extinct-in-the-wild species, the sihek (Guam kingfisher, *Todiramphus cinnamominus*). We then projected population growth under different management alternatives with our *B* estimates incorporated, as well as without inbreeding depression (*B* = 0) or with a conventional default *B*. We found that inbreeding depression severely impacted multiple life-history stages, and directly translated into an effect on population viability under management alternatives. Simulations including our *B* estimates indicated rapid population decline, whereas projections without inbreeding depression or with default *B* suggested very gradual population decline. Further, our results demonstrate that incorporation of *B* across life-history stages can influence management decisions, as projections with our *B* estimates suggested a need to switch to increased breeding management to avoid species extinction and support wild releases. Our results demonstrate that magnitude of *B* across life-history stages can translate into demographic consequences, such that incorporation of multiple life-stage *B* into population models can be important for informed conservation management decision-making.

## Introduction

Threatened species often exist in small, isolated populations and can experience unavoidably high levels of inbreeding that can lead to reductions in fitness trait values termed inbreeding depression^1,2^. Populations that experience high levels of inbreeding have also been shown to have reduced population growth rates and viability (e.g.^3,4^). However, whether detected effects of inbreeding depression on fitness traits directly translate into effects on population growth rate and viability remains controversial^5–7^. The magnitude of inbreeding depression expressed across life-history stages is therefore rarely quantified and incorporated into population projections of management alternatives for threatened species, despite such lack of incorporation potentially risking poor management decisions^8,9^.

The magnitude of inbreeding depression expressed may vary across fitness traits and life-history stages for different species^10–12^. Studies of the impact of inbreeding depression (*B*, number of haploid lethal equivalents) often focus on only parts of the life-cycle and often on early-life stages^1,13,14^. However, inbreeding depression may also impact adult life-history stages (e.g.^15,16^). Further, studies of inbreeding depression across multiple life-history stages have found a cumulative effect, such that the more life-history stages accounted for the greater total *B*^10,17^. Indeed, an estimate of the impact of inbreeding depression on total fitness (i.e. survival to sexual maturity plus adult lifetime reproductive success) in wild song sparrows (*Melospiza melodia*) was substantial (*B*≈25^18^). To build a full picture of impacts of inbreeding on fitness, studies that assess inbreeding depression across multiple life-history stages are therefore required.

For detected effects of inbreeding depression to impact population viability, magnitude of *B* on different fitness traits must translate into an impact on population growth rate^6^. However, studies that quantify *B* on fitness traits often infer an effect on overall population viability, rather than incorporate *B* estimates into population models to directly estimate effect on projected population growth rate (e.g.^19–21^). This difference is important because population growth rate can vary in its sensitivity to changes in different vital rates^22^. Detected impacts of inbreeding depression across life-history stages may therefore not necessarily directly translate into appreciable impacts on population viability (e.g.^7^).

The link between *B* across life-history stages and population viability is especially important in species recovery planning, as projections of population growth rate under management alternatives are often used to evaluate management options (i.e. population viability analyses, PVAs^23,24^). However, PVAs for threatened species often either ignore the impact of inbreeding depression or use default *B* values, as opposed to evaluating and incorporating species-specific impacts of inbreeding depression into population models^8,9,25^. Default *B* values in PVAs are often drawn from studies of parts of the life-cycle (e.g. VORTEX default *B* = 3.145, the combined mean *B* on fecundity and first year survival from^26^,^27^), and are therefore likely to underestimate total impacts of inbreeding depression. For normally outbreeding species that are at risk of inbreeding depression, studies that do not include consideration of inbreeding depression or use defaults drawn from parts of the life-cycle therefore risk over-estimating population viability^8,9,25^, potentially resulting in poor management choices.

Robust estimates of population viability under management alternatives are particularly key for managing extinct-in-the-wild species, where ex-situ (e.g. zoo-managed) populations are critical to ensuring species persistence as well as species recovery. However, species recovery requires harvesting the ex-situ population to re-establish wild populations, but harvests risk jeopardising the ex-situ population’s viability, and thus species persistence if wild populations do not establish. Further, genetic threats may be a particular concern for these species because ex-situ populations are often founded from small numbers of individuals and may have subsequently remained at small size due to limited capacity in breeding facilities. As such, ex-situ populations often experience unavoidably high inbreeding levels, even if best practice genetic management is employed^28^. Further, reductions in inbreeding levels and increases in genetic diversity through supplementation with new wild-caught individuals is not possible. Conversely, *B* is expected to be reduced in benign environments, such as those experienced ex-situ^29,30^. Quantification of *B*, and incorporation of such estimates into population projections of management alternatives, could therefore be highly informative for decision-making and recovery planning of extinct-in-the-wild species.

**Figure 1:**
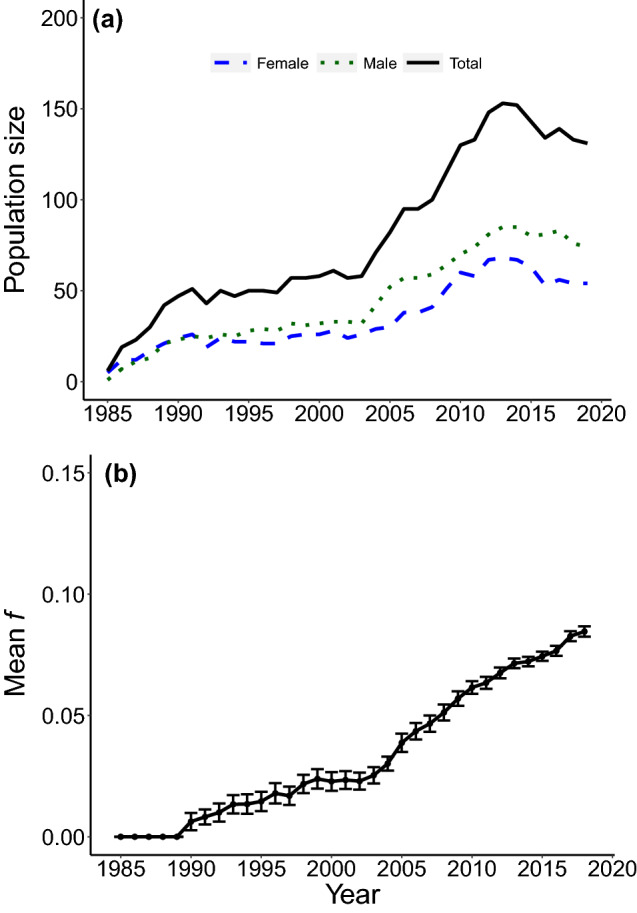
Census size and inbreeding level through time of the sihek ex-situ population. (**a**) Number of ex-situ hatched individuals in the population and (**b**) mean population inbreeding level (*f*), with standard error bars.

One such extinct-in-the-wild species is the sihek or Guam kingfisher (*Todiramphus cinnamominus*). Sihek are endemic to the island of Guam, a United States territory in the Western Pacific^31,32^. The accidental introduction of brown tree snakes (*Boiga irregularis*) to Guam in the 1940’s resulted in extirpation of sihek along with most of Guam’s other forest birds^33,34^. From 1984 to 1986, 29 wild sihek were captured to found the ex-situ population, and sihek were extinct in the wild by 1988^35^. The ex-situ sihek population has subsequently been managed across breeding facilities in the US mainland and in Guam, constituting ~ 140 individuals across 25 institutions in 2019 (Fig. 1a, Supplementary Appendix S1). Current species recovery planning involves consideration of conservation translocations for release of sihek back into the wild. There is therefore a pressing need to assess the viability of the ex-situ population and its ability to support harvests for wild releases.

Here, we estimate *B* across multiple life-history stages for the sihek ex-situ population. We then incorporate our *B* estimates into PVA to directly evaluate the demographic consequences of multiple life-stage inbreeding depression on population viability under alternative breeding management strategies, with and without harvests for release to the wild. Further, we repeat our simulations with *B* = 0 (i.e. without inbreeding depression) and with a conventional default *B* value in PVA, to investigate if inclusion of our *B* estimates would result in different projected population trajectories, and thus different conclusions on viability under management alternatives. Our study provides a rare example of directly translating *B* into effects on population viability under management alternatives, and shows that incorporation of the appropriate *B* value can influence management decision-making. Further, our study is of critical importance to inform sihek conservation, where management objectives include minimizing risks to ex-situ population viability from harvesting, at least until recovery in the wild has been achieved.

## Materials and methods

### Estimation of inbreeding and inbreeding load

To quantify impact of inbreeding depression across different life-history stages, we investigated the phenotypic traits of first-year survival (from hatching to age 1), second-year survival (age 1 to age 2) and adult (age 2 +) female and male longevity, as well as breeding success.

Individual inbreeding coefficients (*f*) were calculated from pedigree data for all birds in the ex-situ population up to February 2019, from the North American Regional Guam Kingfisher Studbook, using PMx v1.6^36^. Of the birds initially captured from the wild, only 16 founders contributed genetically to the current population^37^. Of these founders, minisatellite DNA profiles revealed likely full-sibling relationships between two pairs of founders^35^. These individuals were therefore assigned as full-siblings in the species’ studbook, such that these relationships were accounted for in subsequent breeding plans and pedigree analyses. All other founders were assumed unrelated in the pedigree; *f* of individuals in subsequent generations are relative to the founder population.

Data from captive-hatched individuals from 1985 to 2018 were included in models for survival and longevity analyses. Models of the effect of individual *f* on first-year and second-year survival were fitted using generalized linear models (GLMs). Both the male and female sihek contribute to provisioning offspring, therefore sire and dam quality may affect first-year survival^31,32^. Further, ex-situ sihek hatchlings may be parent-reared or removed for hand-rearing. Models of first-year survival therefore included maternal and paternal *f* and rearing type (i.e. hand or parent-reared) as explanatory variables. Individual’s sex was not included as sihek are sexed using plumage sexual dimorphism which develops around fledging age (~ 30 days post-hatch) and most first-year mortality occurred pre-fledging. As sihek are moved from being housed with parents within 2–3 months post-hatch, models of second-year survival did not include parent effects but did include individual’s sex and individual *f* as explanatory variables. The potential effect of different breeding facilities was explored in models of both first- and second-year survival by including the facility that each individual was located at as a random effect in generalized linear mixed effects models (GLMMs), however estimates of variance across facilities was estimated to be zero so this term was removed from final models.

Models of the effect of individual *f* on adult longevity were fitted using GLMMs, with breeding facility included as a random effect because variance across facilities for this trait was > 0. Models were fitted separately for males and females as maximal age has been observed to be substantially different between the sexes in captivity (oldest male and female recorded were 23 and 15 years respectively).

The effect of parents’ *f* on reproductive success was examined as the number of hatchlings produced over the duration that a breeding pair was together. Reproductive success was considered at the breeding pair level because genetic management of the population is carried out through selection and management of breeding pairs, based on individuals’ mean kinship and pairs’ kinship coefficient, *k*^28^ (Supplementary Appendix S1). Therefore, to account for differences in management between pairs, explanatory variables considered were dam and sire’s individual mean kinships at year of pairing and each pairs’ *k*. Additionally, dam and sire’s *f,* pair duration and pair duration-squared and dam age at pairing and age-squared were considered as explanatory variables. Squared age and pair duration terms were considered to explore potential non-linear relationships. Models were fitted using GLMMs, with breeding facility and dam and sire identities included as random effects as pairs may be split and individual’s re-paired, such that they may contribute to more than one pairing. Data from ex-situ hatched individuals from 1985 to 2014 was used for this analysis, as information on pair duration was not readily available for later years, and only data from completed pairings (i.e. the pair had been split or one individual of the pair had died) were used.

Collinearity between explanatory variables was checked by calculating Pearson correlations and variance inflation factors (VIFs). Year of study was found to be strongly correlated with individual *f* (Pearson’s *r* = 0.74), and resulted in a high variance inflation factor (VIF > 10), as expected given the small, closed nature of the sihek population such that relatedness of individuals, and therefore also mean population *f*, will inevitably increase with time (Fig. 1b). Collinearity can result in unreliable parameter estimates^38^, and therefore year of study was removed from models. Models of first-year survival and reproductive success were initially fitted with all variables potentially explaining variation in the trait of interest. Model selection was then performed by sequentially removing least significant terms in the model and comparing model fit using Akaike’s information criterion (AIC,^39^), but with individual’s *f* retained in all models. For models of reproductive success, as this trait encompassed both production of eggs and survival to hatching, pair’s *k* (which is equal to offspring *f*) was also retained in the model despite non-significance to account for any inbreeding depression in offspring’s survival to hatch. As selection of a single ‘best’ minimum adequate model with which to base inference on can result in potentially misleading conclusions^40^, parameter estimates from our final models were compared with estimates from our maximal models to check consistency of inference (Supplementary Appendix S2).

All models were fitted with Poisson error distributions and log link functions to ensure comparable estimates of *B*, following^41^. When applying Poisson regression to binomial data, error and therefore width of confidence intervals may be overestimated. Robust standard errors from models of first- and second-year survival were therefore calculated using the sandwich estimator^42^. GLMs were fitted in base R and GLMMs were fitted using the R package lme4^43^. For models of reproductive success, the bobyqa optimizer was used to assist model convergence by increasing maximum iterations. All statistical analyses were carried out in R 3.5.3^44^.

### Population viability analysis

To evaluate ex-situ sihek population viability under alternative management strategies, we used an individual based population model. This modelling approach allows incorporation of our *B* estimates with associated uncertainty, as well as variation in individual *f,* and demographic and environmental stochasticity.

**Figure 2:**
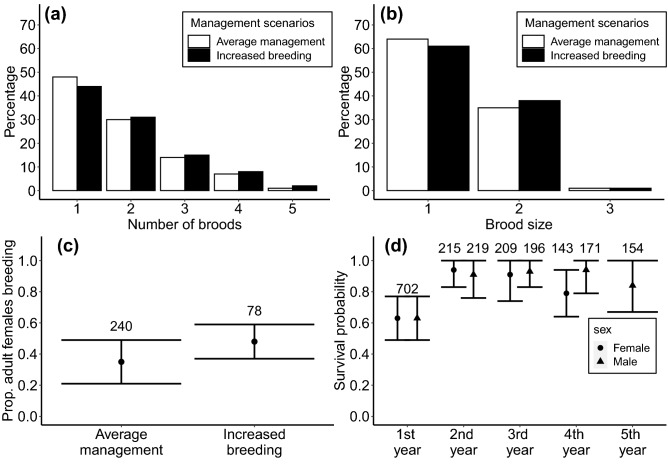
Demographic rates from the sihek ex-situ population used in population models of management scenarios. (**a**) Number of broods produced and (**b**) brood size given breeding, (**c**) proportion of adult females breeding per year (i.e. producing ≥ 1 hatchling) with associated standard deviations, and (**d**) sex-age class survival probabilities with associated standard deviations. In (**d**), females and males were pooled for estimation of first-year survival probability. Female fourth-year survival probability represents survival probability of females aged 3 +, male fifth year survival probability represents survival probability of males aged 4 +. For (**c**) and (**d**) associated sample sizes are shown above each error bar.

We first built a baseline population model, parameterised from studbook records from captive-hatched individuals from 1985 to 2018, to reflect the ex-situ population over the course of the sihek breeding program to date. Full details of parameter estimation and modelling are provided in Supplementary Appendix S3. Age at first breeding was modelled to occur at the median age of first hatchling production to best reflect population dynamics^27^, estimated as from age three and four for females and males, respectively. Reproductive rates were estimated from the average proportion of captive-hatched adult females that successfully produced at least one hatchling each year, the number of broods produced per year (with a brood being defined as hatchlings produced within a seven-day period), and the distribution of brood sizes produced (Fig. 2a–c). As sihek in the ex-situ population are monitored daily, hatch and death dates are known with high accuracy. Sex-age class survival probabilities (*ϕ*_*s*_), and hence mortality rates, were estimated from studbook data for individuals hatched from 1985 to 2018. Male and female hatchlings were pooled for calculation of first-year survival probability (*ɸ*_*1*_) because most individuals that died in their first year died before age at sexing. Age-specific survival probabilities from age one onwards were estimated for males and females separately, so that the model was structured with separate sex-age classes up to age two for females and age three for males (i.e. the median sex-specific age at first breeding), then with an adult female (*ɸ*_*F*_) and male (*ɸ*_*M*_) stage class representing females aged 3 + and males aged 4 + (Fig. 2d, Table S4). Observed sex-ratios of nestlings at age of sexing was not significantly different from 1:1 (240 females, 251 males, χ^2^ = 0.25, df = 1, p-value = 0.62), therefore simulated offspring were assigned as female or male with equal probability.

We modelled initial population *f* as the mean *f* of all individuals alive in February 2019, to reflect the current population. Individual *f* of modelled individuals in subsequent generations were calculated from the simulated pedigree using standard algorithms^27^. Our significant (i.e. 95% confidence intervals did not overlap zero) estimates of *B* on reproductive success and adult female and male longevity were modelled through reductions in *ɸ*_*1*_, *ɸ*_*F*_ and *ɸ*_*M*_, respectively (Supplementary Appendix S4). Our estimate of *B* for reproductive success represents the combined trait of production of eggs and offspring survival to hatch, and is likely to be reasonably well captured by applying entirely to modelled *ɸ*_*1*_ (Lacy, et al., 2017). Our *B* estimates had relatively high associated uncertainties, which we explicitly incorporated into our model by each model replicate drawing *B* from a Gaussian distribution with our point estimates as the mean and their standard error as the standard deviation.

### Modelled scenarios

We modelled four scenarios of (1) average management and (2) increased breeding management, as well as harvests under (3) average management and (4) increased breeding management.

Management recommendations concerning breeding of sihek have changed through the course of the breeding program. Our average management scenario was based on the average reproductive rates (i.e. proportion of adult females breeding and distributions of number of broods produced and brood sizes given breeding) achieved across the full ex-situ breeding program, estimated from captive-hatched individuals from studbook records from 1985 to 2018 (i.e. the ‘baseline model’, Fig. 2a–c, Supplementary Appendix S3). Our increased breeding management scenario was formulated in consultation with the sihek conservation translocation planning working group, including the sihek Species Survival Plan (SSP) coordinator and was based on the reproductive rates achieved during the period of increasing population size from 2004 to 2013 (Fig. 1), when recommendations to increase breeding for the sihek SSP were issued (Fig. 2a–c, Supplementary Appendix S3). Under all management scenarios, simulated pairs were selected based on mean kinship at each time step to reflect the best-practice genetic management of the ex-situ population, with pairs retained for three years, before being split and re-paired (Supplementary Appendix S1).

Our harvesting scenarios (iii–iv) were designed to reflect potential harvesting of individuals for small trial releases to the wild, from the ex-situ population under our management scenarios (i–ii). As such, we modelled harvests of ten age-one individuals removed annually from years 2–6, with an equal sex ratio being harvested, applied to our modelled scenarios of (i–ii).

To investigate the effects of not accounting for inbreeding depression or using a conventional default *B* value on model projections, we repeated our four management scenarios without inbreeding depression (i.e. *B* = 0) and using the default value of 2*B* = 6.29 (i.e. *B* = 3.145) used in VORTEX v10.2.17.0^45^ applied to *ɸ*_*1*_.

### PVA model implementation and analysis

The population model was built in program VORTEX v10.2.17.0^45^. Full model input parameters are specified in Supplementary Appendix S4. Starting modelled population size and age distribution were set to reflect the ex-situ population in 2019. Carrying capacity was set to the target population size as specified in the sihek SSP of 200 individuals (Supplementary Appendix S3).

Since we were interested in effects of inbreeding that may impact population viability over relatively short time-frames (Keller and Waller 2002) but sihek have moderately long generation times (~ 6 and ~ 8 years for females and males, respectively), we defined a 50 year time-frame for our PVA to encompass ~ 6–8 sihek generations. Each model was replicated 5000 times. To compare the focal management scenarios, for each replicate we extracted population size in year 50 and mean stochastic population growth rate ($\overline{\lambda }_{s}$) calculated as the mean of the growth rate across extant years. Means and standard deviations for these metrics were calculated across replicates, using R 3.5.3^44^.

### Ethical statement

Data used in this study was obtained from the North American Regional Guam Kingfisher Studbook, which is maintained as part of the sihek breeding program. No experiments were carried out on birds for this study.

## Results

### Inbreeding and inbreeding load

Across 702 captive-hatched sihek between 1985 and 2018, individual *f* varied from 0.00 to 0.25 (median *f* = 0.07, variance = 0.002, Fig. S1 Supplementary Appendix S2). Mean population *f* increased but variance decreased across years of the breeding program (Fig. 1b).

We found no significant effect of individual *f* on first-year survival (*B* = − 0.67, 95% CI − 1.34 to 2.70, *p* = 0.684) or second-year survival (*B* = − 6.77, 95%CI − 3.83 to 17.37, *p* = 0.209), suggesting no detectable inbreeding depression in these traits (Supplementary Appendix S2). We also found no significant effect of sire’s *f* on first-year survival (sire *f*: estimate = 2.80 ± 1.13SE, *p* = 0.146). Rearing type had a strong effect on first-year survival, with hand-reared hatchlings being significantly more likely to survive to age one than parent-reared hatchlings (estimate = − 0.55 ± 0.08SE, *p* < 0.001). Dam *f* was non-significant and inclusion did not improve model fit, so this term was removed from the final model (Supplementary Appendix S2). To test whether individuals with high *f* relative to the rest of the population influenced results, we repeated analysis of first-year survival excluding one individual with *f* = 0.25 (Fig. S1 Supplementary Appendix S2). Removal of this individual did not change inference, with no significant effect of individual *f* on first-year survival (*B* = − 0.27, 95% CI − 1.76 to 2.31, *p* = 0.88). This individual died in its first year and therefore was not included in models of the effect of individual *f* on our other investigated traits.

We found a significant effect of individual *f* on adult male and female longevity (adult male: *B* = − 5.54, 95% CI − 7.47 to − 3.63, *p* < 0.001; adult female: *B* = − 2.82, 95% CI − 4.65 to − 0.98, *p* = 0.003), suggesting significant inbreeding depression in this trait for both sexes, but with a greater impact on adult male than female longevity (Supplementary Appendix S2).

Across 114 unique pairs formed from 69 sires and 63 dams, we found a significant effect of dam’s *f* on number of hatchlings produced (*B* = − 8.43, 95% CI − 15.17 to − 1.68, *p* = 0.014), suggesting significant inbreeding depression in female reproductive success across the pair’s duration (Supplementary Appendix S2). The effect of sire’s *f* on number of hatchlings produced was marginally non-significant and positive, such that there was no evidence of inbreeding depression in male reproductive success (*B* = 7.83, 95% CI – 0.24 to 15.90, *p* = 0.057). We found no significant effect of pair’s *k*, and thus hatchling’s *f*, on number of hatchlings produced, suggesting no detectable inbreeding depression in early-life survival to hatching (*B* = 1.08 ± 2.71SE, *p* = 0.69). There was a strong positive effect of pair duration on female reproductive success as well as a marginally non-significant effect of pair duration-squared, suggesting that pairs that are together for longer tend to produce more offspring but offspring production may slow over time (pair duration: estimate = 0.80 ± 0.15SE, *p* = 0.001; pair duration-squared: estimate = − 0.07 ± 0.02SE, *p* = 0.092). Mean kinship terms were non-significant and inclusion did not improve model fit, so these terms were removed from the final model. In reality, our final and maximal models resulted in similar parameter estimates (Supplementary Appendix S2). Further, as incorporation of our *B* estimates within PVAs explicitly accounted for parameter uncertainty, maximal model *B* fell within the specified distribution of our modelled *B* for reproductive success.

### Population viability analysis

**Figure 3:**
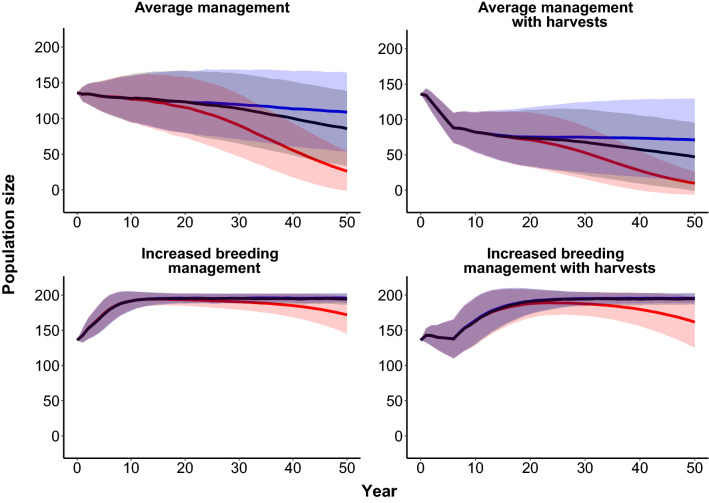
Simulated size of the sihek ex-situ population under alternative management scenarios. Solid lines indicate mean population size across model iterations, with standard deviation (shaded areas). Colours indicate model scenarios including our sihek *B* estimates (red), no inbreeding depression (blue), or default *B* (black).

With our *B* estimates incorporated into our population model, the sihek ex-situ population is projected to rapidly decline ($\overline{\lambda }_{s}$ = 0.96 ± 0.03SD) under our average management scenario, with harvests substantially increasing rate of decline ($\overline{\lambda }_{s}$ = 0.93 ± 0.03SD, Fig. 3). However, when we repeated our average management scenario without inbreeding depression or with default *B*, our projections indicated a gradual population decline ($\overline{\lambda }_{s}$ = 0.99 ± 0.01 SD, Fig. 3), and underestimated the projected rate of population decline when harvesting was added (without inbreeding depression: $\overline{\lambda }_{s}$ = 0.98 ± 0.02 SD; with default *B*: $\overline{\lambda }_{s}$ = 0.97 ± 0.02 SD, Fig. 3).

In contrast, under our scenario of increased breeding management and including our *B* estimates, the population is projected to increase to carrying capacity ($\overline{\lambda }_{s}$ = 1.01 ± 0.02SD, Fig. 3), with simulations of harvests resulting in a similar population trajectory ($\overline{\lambda }_{s}$ = 1.01 ± 0.02SD, Fig. 3). When we repeated our increased breeding management scenario with and without harvests and without inbreeding depression or with default *B*, projections indicated a slight overestimation of the rate of population increase (without harvests and no inbreeding depression or default *B*: $\overline{\lambda }_{s}$ = 1.02 ± 0.01 SD; with harvests and no inbreeding depression or default *B*: $\overline{\lambda }_{s}$ = 1.02 ± 0.01 SD, Fig. 3).

## Discussion

Whether magnitude of inbreeding depression detected across fitness traits can directly translate into impacts on population viability, and thus influence management decisions, is a key research question in applied ecology^5,6^. We assessed inbreeding depression across life-history stages for sihek and found substantial inbreeding depression in adult life-history traits, but no detectable inbreeding depression in juvenile traits. When incorporated into our population model, these impacts of inbreeding depression influenced projected viability and suggest a need to switch management strategies to increase breeding, to ensure long-term ex-situ population viability and support harvests for wild releases. Importantly, our results suggest different conclusions on viability under management alternatives could have been drawn if a commonly used default *B* or no inbreeding depression had been used in population projections.

### Inbreeding load across life-history stages

We detected substantial inbreeding depression in reproductive success (*B* ~ 8.4) and in adult male longevity (*B* ~ 5.5), and lower but still significant inbreeding depression in adult female longevity (*B* ~ 2.8). In contrast, we found no detectable inbreeding depression in first- or second-year survival. This finding in sihek contrasts with a study across multiple captive mammal populations, that found mean *B* ~ 2.3 for juvenile survival, although with a wide range of *B* = − 0.7 to 15.2^13^. Strength of inbreeding depression in single life-history stages covering short time-spans may be expected to be lower, and thus more difficult to detect, than for multi-year adult life-history traits as inbreeding depression accumulates across the life-span^11^. Further, management strategies such as hand-rearing hatchlings, which was significantly associated with increased first year survival, may act to mask inbreeding depression and thus also make it more difficult to detect. Deleterious recessive alleles are often expressed during early development^46,47^, thus inbreeding depression could be expressed at pre-hatch life stages in the sihek. However, in our models of reproductive success (which represent the combined trait of pair’s egg production and egg survival to hatch), pair’s *k* and thus egg *f* were found to be non-significant, suggesting no detectable inbreeding depression at the pre-hatch stage. Alternatively, *B* may truly be negligible in sihek juvenile survival as juvenile traits may be subject to strong selection and therefore may have been previously purged of deleterious alleles^48,49^. Our results highlight the need to quantify inbreeding depression across life-history stages, as opposed to focusing on juvenile life-history stages.

Our *B* estimate in adult male longevity was substantially higher than that for adult female longevity. Sex-specific differences in inbreeding depression have been found in a variety of species, including in captive canaries (*Serinus canaria*,^50^) as well as wild song sparrows^51^. Senescence theory predicts that individuals that live longer will experience greater inbreeding depression due to weaker selection against late-acting deleterious alleles^52,53^. Adult male survival probability was higher than adult female’s in the sihek ex-situ population (Fig. 2d). Our observed sex-specific differences in *B* on adult longevity is therefore consistent with sex-specific mortality observed in sihek.

Ex-situ populations are expected to exhibit relatively low inbreeding depression due to the benign environmental conditions they inhabit^29,54^. Our estimates on reproductive success and adult longevity are relatively high compared to other ex-situ populations, e.g. *B* ~ 5.4 in reproductive success of wolves (*Canis lupus,*^55^) and *B* ~ 0.87 and 0.82 in longevity of adult male and female butterflies (*Bicyclus anynana*,^16^). Our estimates are also relatively high compared to some wild populations for reproductive success (e.g. *B* ~ 3.1 in reproductive success of great tits, *Parus major*,^17^) and comparable for adult longevity (e.g. *B* ~ 6.3 in adult longevity in white-footed mice, *Peromyscus leucopus*,^15^), despite wild environmental conditions being expected to be more stressful and thus inbreeding depression more severe^29,30^. Further, our estimates are relatively high despite likely historical population bottleneck events (Supplementary Appendix S1), which may have presented opportunities for purging of genetic load^56^. Our results add to the substantial variation in estimates of magnitude of inbreeding depression detected across species^41,56^. Further, they suggest relatively high inbreeding depression can occur in benign ex-situ environments, thus highlighting the need to consider genetic threats to populations of conservation concern even in ex-situ environments.

Due to the small size and insularity of the sihek population, individual *f* and year of study were collinear, and therefore year of study was excluded from our estimations of *B* to avoid variance inflation and unreliable parameter estimates^38^. Fitness trait values may be expected to increase through time for ex-situ sihek, as husbandry techniques improve (e.g.^57^) or adaptation to ex-situ conditions occurs (e.g.^58,59^). Such effects would be expected to act in the opposite direction to our observed effects of inbreeding depression, thus potentially reducing our ability to detect inbreeding depression and downwardly biasing *B* estimates. Further, variance in *f* in our study was relatively low (variance = 0.002) compared to in other pedigreed populations (e.g. median variance = 0.0031 across a variety of mammals and birds,^60^), therefore reducing power to detect inbreeding depression. Our *B* estimates may therefore represent underestimates of true inbreeding depression in this population.

### Population viability and management decision-making

A previous study that detected inbreeding depression on female fecundity in bighorn sheep (*Ovis canadensis sierrae*) found that detected effects had little impact on overall population growth rate^7^. In contrast, our detected impacts of inbreeding depression across life-history traits did directly translate into an effect on population viability. Under average management, with our *B* estimates incorporated, simulations suggested the sihek population is projected to rapidly decline, whereas simulations without inbreeding depression suggested only a very gradual population decline. Further, our *B* estimates translated into an effect on management decision-making, because projections with our *B* estimates incorporated suggested a switch from average management to increased breeding management is required to achieve population viability and allow sustainable harvests for releases to the wild. Our results therefore demonstrate that detected impacts of inbreeding depression can have demographic consequences, such that they can influence population viability and management strategies for threatened species.

PVAs of management alternatives often use the default 2*B* value of 6.29 applied to first year survival (e.g.^61–63^). When we repeated our simulations with this default *B* value, our projections resulted in the same over-estimation of population growth rate as with no inbreeding depression included. Such over-estimation of $\overline{\lambda }_{s}$ risks leading to poor management decisions. Our results suggest magnitude of *B* across life-history stages included in PVAs can influence projected outcomes across management alternatives, such that incorporation of multiple life-stage *B* in population models could assist rational decision making. Where possible, this requires estimation of *B* across multiple life-history stages for the focal population. For ex-situ and wild populations with detailed individual-based monitoring, *B* may be estimated using available monitoring data. For other populations calculation of *B*, although not trivial, is increasingly attainable through use of genomic methods such as ‘runs of homozygosity’ to estimate *f*^5,64^. Where not possible, *B* estimates from other systems that use unbiased statistical models^41^ and which consider multiple life-history stages, such as presented in this study, could be used.

Our projections do not take into account potential purging of deleterious alleles into the future, which could mitigate impacts of inbreeding depression^65^, such that long-term $\overline{\lambda }_{s}$ could be higher. However, purging may be ineffective in captivity, where benign conditions offer little opportunity for selection^2^. Establishment of a wild population could allow more opportunity for purging^1^. However, inbreeding depression may be expected to be stronger in harsh wild environments^29,30^, potentially leading to increased extinction risk for any reintroduced wild populations^66^.

For sihek, our results clearly emphasize the need for increased breeding to avoid imminent decline of the ex-situ population and therefore the species. The sihek ex-situ population has been at reduced size (≤ 157 individuals) for > 30 years (Fig. 1a). Due to its small size and insularity, further increases in *f* and associated expression of inbreeding depression in the sihek population are inevitable, but rate of increase can be minimized by continuation of best-practice genetic management. Further, our scenario of increased breeding suggests such management could at least partly compensate for impacts of inbreeding depression and result in increasing population size. We encourage management to increase current sihek population size, to reduce vulnerability to demographic stochasticity and genetic threats associated with small population size^1,67^. We recognise that this may require new breeding institutions and has to be balanced against cost and other competing conservation objectives of host institutions.

Overall, our results provide evidence that detected impacts of inbreeding depression on fitness traits can directly translate into demographic consequences. Further, our study suggests that magnitude of inbreeding depression considered across life-history stages can affect overall population growth rate and viability under management alternatives, such that incorporation of multiple life-stage *B* into population models can be highly informative for management decision-making for threatened species. This is particularly pertinent in conservation planning for extinct-in-the-wild species, where management decisions must not jeopardise ex-situ population viability and thus species persistence.

## Supplementary Information

Supplementary Information

## Data Availability

Data associated with this article are available upon reasonable request to the authors.
