# Supplementary material for: Multiple life-stage inbreeding depression impacts demography and extinction risk in an extinct-in-the-wild species
Source: Sci Rep. 2021 Jan 12;11:682. doi: 10.1038/s41598-020-79979-4 (PMC7804286; doi:10.1038/s41598-020-79979-4)
Supplement: Supplementary file 1 — Supplementary Information [file 41598_2020_79979_MOESM1_ESM.docx]

***Supplementary material for:***

**Multiple life-stage inbreeding depression impacts demography and extinction risk in an extinct-in-the-wild species**

**Trask, A.E., Ferrie, G.M., Wang, J., Newland, S., Canessa, S., Moehrenschlager, A., Laut, M., Barnhart Duenas, L., Ewen J.G**

**Contents:**

Supplementary Appendix 1. Focal population

Supplementary Appendix 2. Models of inbreeding load (*B*) on sihek life-history traits

Supplementary Appendix 3. Parameter estimation for population viability analysis

Supplementary Appendix 4. Population viability analysis - model input parameter values

**Supplementary Appendix 1. Focal population**

Sihek are likely to have experienced historical population bottleneck events: during their initial colonization of Guam, their decline following introduction of brown tree snakes (*Boiga irregularis*), the founding of the *ex-situ* population, and during the early years of the breeding program when not all founders bred successfully ^1,2^. These sequential bottlenecks likely resulted in loss of genetic diversity through drift ^2^, but also increased inbreeding levels that could have resulted in previous purging of inbreeding depression ^3^. The sihek *ex-situ* breeding program was initiated in 1984, with the first zoo-hatched chicks occurring in 1985. The population subsequently increased to 61 birds by 1990 and remained at around this size until 2004 (Fig. 1). Over the following 10 years, the population increased to a peak of 157 birds, in response to calls to increase *ex-situ* breeding efforts to increase the population size. The population size in 2019 was ~140 birds, across 25 institutions. *Ex-situ* breeding of sihek is managed through the Guam kingfisher (sihek) Species Survival Plan (SSP), through the Association of Zoos and Aquariums (AZA) and in partnership with Guam Division of Aquatic and Wildlife Resources (DAWR).

The sihek population is managed to retain maximal genetic diversity and prevent close inbreeding through careful selection of breeding pairs, based on mean kinship (i.e. the mean of the kinship coefficients between that individual and all living individuals in the population), and avoiding pairs that would produce offspring with high *f* relative to the rest of the population. Further, breeding pairs are managed, such that pairs deemed to be genetically important based on mean kinships may be prioritized for breeding ^4^. Sihek are socially monogamous and form long-term, highly territorial breeding pairs in the wild ^5,6^. Breeding pairs are therefore housed in separate enclosures and kept together for at least three years after laying their first egg, before being re-paired. Breeding pairs are monitored, and number of broods produced, number of hatchlings per brood, as well as subsequent survival, recorded.

**Supplementary Appendix 2.** **Models of inbreeding load (*B*) on sihek life-history traits**

**Table S1.** Final models investigating the effects of inbreeding on pair reproductive success, first- and second-year survival, and adult male and female longevity. Estimated effect sizes (*B*) for all fixed effects with standard errors, SE (SE’s for models of 1^st^- and 2^nd^- year survival were estimated using the sandwich estimator^7^), and variance explained by random effects with standard deviations (SD). Variance explained by each model (coefficient of determination, *R^2^*) and sample sizes (*N*) are shown.

| **Modelled trait:** | ***Fixed effect*** | ***B*** | ***SE*** | ***Z*** | ***P*** | ***R^2^*** |
| --- | --- | --- | --- | --- | --- | --- |
| **Pair reproductive success** | Intercept | -2.03 | 0.76 | -2.67 | 0.008 | 0.74† |
| ***N*=114** | Dam’s *f* | -8.43 | 3.44 | -2.45 | 0.014 |  |
|  | Sire’s *f* | 7.83 | 4.12 | 1.90 | 0.057 |  |
|  | Pair’s *k* | 1.08 | 2.71 | 0.40 | 0.690 |  |
|  | Dam’s age | 0.25 | 0.24 | 1.67 | 0.095 |  |
|  | Dam’s age^2^ | -0.03 | 0.04 | -1.65 | 0.098 |  |
|  | Pair duration | 0.80 | 0.15 | 3.28 | 0.001 |  |
|  | Pair duration^2^ | -0.07 | 0.02 | -1.69 | 0.092 |  |
|  | ***Random effect*** | ***Variance*** | ***SD*** |  |  |  |
|  | Dam’s ID | 0.09 | 0.30 |  |  |  |
|  | Sire’s ID | 0.30 | 0.55 |  |  |  |
|  | Location | 3.77 | 1.94 |  |  |  |
| **1^st^ year survival** | Intercept | -0.33 | 0.05 | -3.88 | 0.001 | 0.09‡ |
| ***N*=687** | Individual’s *f* | -0.67 | 1.03 | -0.41 | 0.684 |  |
|  | Sire’s *f* | 2.80 | 1.13 | 1.45 | 0.146 |  |
|  | Rearing type | -0.55 | 0.08 | -4.98 | <0.001 |  |
| **2^nd^ year survival** | Intercept | 2.87 | 0.41 | 7.21 | <0.001 | 0.02‡ |
| ***N*=317** | Individual’s *f* | -6.78 | 5.41 | -1.26 | 0.209 |  |
|  | Sex | -0.58 | 0.40 | -1.45 | 0.147 |  |
| **Adult male longevity** | Intercept | 2.14 | 0.07 | 31.13 | <0.001 | 0.43† |
| **N=140** | Individual’s *f* | -5.54 | 0.98 | -5.68 | <0.001 |  |
|  | ***Random effect*** | ***Variance*** | ***SD*** |  |  |  |
|  | Location | 0.06 | 0.25 |  |  |  |
| **Adult female longevity** | Intercept | 1.91 | 0.05 | 36.62 | <0.001 | 0.11† |
| **N=163** | Individual’s *f* | -2.82 | 0.94 | -3.01 | 0.003 |  |
|  | ***Random effect*** | ***Variance*** | ***SD*** |  |  |  |
|  | Location | 0.01 | 0.09 |  |  |  |

†*R^2^* values for generalised linear mixed effects models are conditional *R^2^*, which represents the variance explained by the fixed and random effects together, and were estimated following ^8^ in the R package Performance ^9^.

‡ *R^2^* values for generalised linear models were estimated using Nagelkerke’s *R^2^* in the R package DescTools ^10^.

**Table S2.** Maximal models (i.e. including all explanatory variables of interest) investigating the effects of inbreeding on pair reproductive success and first-year survival. Estimated effect sizes (*B*) for all fixed effects with standard errors, SE (SE’s for the model of 1^st^- year survival were estimated using the sandwich estimator^7^), and variance explained by random effects with standard deviations (SD). Variance explained by each model (coefficient of determination, *R^2^*) and sample sizes (*N*) are shown.

| **Trait** | ***Fixed effect*** | ***B*** | ***SE*** | ***Z*** | ***P*** | ***R^2^*** |
| --- | --- | --- | --- | --- | --- | --- |
| **Pair reproductive success**  ***N*=114** | Intercept | -2.82 | 0.89 | -3.16 | 0.002 | 0.81† |
|  | Dam’s *f* | -8.60 | 4.32 | -1.99 | 0.046 |  |
|  | Sire’s *f* | 7.10 | 4.61 | 1.54 | 0.124 |  |
|  | Pair’s *k* | -0.06 | 2.72 | -0.02 | 0.982 |  |
|  | Dam’s age | 0.25 | 0.24 | 1.67 | 0.094 |  |
|  | Dam’s age^2^ | -0.03 | 0.02 | -1.41 | 0.158 |  |
|  | Pair duration | 0.76 | 0.23 | 3.30 | 0.001 |  |
|  | Pair duration^2^ | -0.05 | 0.04 | -1.40 | 0.163 |  |
|  | Dam mean kinship | 3.70 | 5.92 | 0.63 | 0.532 |  |
|  | Sire mean kinship | 0.40 | 4.91 | 0.08 | 0.934 |  |
|  | ***Random effect*** | ***Variance*** | ***SD*** |  |  |  |
|  | Dam’s ID | 0.07 | 0.27 |  |  |  |
|  | Sire’s ID | 0.22 | 0.47 |  |  |  |
|  | Location | 5.92 | 2.43 |  |  |  |
| **1^st^ year survival** | Intercept | -0.35 | 0.09 | -4.13 | <0.001 | 0.09‡ |
| ***N*=687** | Individual’s *f* | -0.78 | 1.89 | -0.41 | 0.680 |  |
|  | Sire’s *f* | 2.34 | 2.03 | 1.15 | 0.251 |  |
|  | Rearing type | -0.53 | 0.11 | -4.97 | <0.001 |  |
|  | Dam’s *f* | 1.15 | 2.24 | 0.51 | 0.608 |  |

†*R^2^* values for generalised linear mixed effects models were estimated following ^8^ in the R package Performance ^9^, and give conditional and marginal *R^2^*, which represent the variance explained by the fixed effects only and fixed and random effects together, respectively.

‡*R^2^* values for generalised linear models were estimated using Nagelkerke’s *R^2^* in the R package DescTools ^10^.

**Table S3**. Akaike’s information criterion (AIC) values of maximal and final models investigating the effects of inbreeding on pair reproductive success and first-year survival. Pair reproductive success models were also fitted with dam and sire ID’s and location random effects.

| **Candidate model** | **AIC** |
| --- | --- |
| **Trait: Pair reproductive success** |  |
| *Maximal model:*  β0 + dam’s *f* + sire’s *f* + pair’s *k*+ dam age+ I(dam age^2) + pair duration + pair duration + I(pair duration^2)+ dam mean kinship + sire mean kinship | 550.6 |
| *Final model:*  β0 + dam’s *f* + sire’s *f* + pair’s *k*+ dam age+ I(dam age^2) + pair duration + pair duration + I(pair duration^2) | 528.5 |
| **Trait: 1^st^ year survival** |  |
| *Maximal model:*  β0 + individual’s *f* + sire’s *f* + dam’s *f* + rearing type | 1191.3 |
| *Final model:*  β0 + individual’s *f* + sire’s *f* + rearing type | 1189.7 |

Fig. S1. Frequency distribution of individual inbreeding coefficients (*f*) of all zoo-hatched individuals in the sihek *ex-situ* population.

**Supplementary Appendix 3. Parameter estimation for population viability analysis**

- 1. *Baseline demography*

The baseline population model was parameterised to reflect the *ex-situ* population over the full course of the sihek breeding program. Reproductive rates were therefore estimated from zoo-hatched individuals from studbook records from 1985-2018. Although sihek have been observed breeding from age one in the *ex-situ* population, age at first breeding was modelled to occur at the median age at production of first hatchling to best reflect population dynamics (Lacy et al., 2017), estimated as from age three and four for females and males, respectively. Reproductive success was modelled in three steps: the probability of each adult female successfully breeding each year (i.e. producing one or more hatchlings); the distribution of number of broods produced per year, conditional on breeding success; and the distribution of brood sizes produced ^11^. Reproductive rates were estimated from the average proportion of *ex-situ* -born adult females that successfully produced at least one hatchling each year, the number of broods produced per year (with a brood being defined as hatchlings produced within a seven-day period), and the distribution of brood sizes produced.

As sihek in the *ex-situ* population are monitored daily, hatch and death dates are known with high accuracy. Age-specific annual survival probabilities, and hence mortality rates, were estimated from studbook data for individuals hatched from 1985-2018 (i.e. excluding wild-caught individuals which were of unknown age). First-year survival probability was estimated as the mean proportion of individuals that hatched that survived to age one across years. Male and female hatchlings were pooled because most individuals that died in their first year died before age at sexing. Age-specific survival probabilities from age one onwards were estimated for males and females separately, so that the model was structured with separate sex-age classes up to age three for females and age four for males (i.e. the median sex-specific age at first breeding), then with an adult female class representing females aged 3+ and an adult male class representing males aged 4+ (Fig 2d main manuscript, Table S4). Sex-age class survival probabilities were estimated as the mean proportion of individuals of each age class that survived to the following age class across years. Female age 3+ and male age 4+ stage class survival probabilities were estimated as the grand mean across age 3 to age 15 (maximum recorded female age) age classes and age 4 to age 23 (maximum recorded male age) age classes for males and females respectively. Mortality of simulated individuals was modelled at each annual time‐step based on *ϕ_s_* for each sex-age class. Among-year environmental variation in *ɸ_s_* and reproductive success, as well as in the correlation between *ɸ_s_* and reproductive success, was assumed to be negligible due to regulation of *ex-situ* environmental conditions, and was therefore set to zero.

- 1. *Modelled scenarios*

Across the full sihek breeding program, the mean number of hatchlings produced per female given breeding per year was 2.54± 0.10 SE (range: 1-10). This increased to 2.70± 0.17 SE hatchlings per female given breeding per year across the years of rapid population growth from 2004-2013 (when recommendations to increase breeding for the sihek SSP were issued).

Our (i) average management scenario was created to capture the average performance of the *ex-situ* population across the full program and encompassing any changes in breeding management recommendations. Our (ii) increased breeding management scenario was created to reflect the productivity the *ex-situ* population may be able to achieve if managers were asked to increase breeding. This scenario was therefore based on what had previously been achieved by managers when asked to increase breeding, and was formulated in consultation with the sihek conservation translocation planning working group, including the sihek Species Survival Plan (SSP) coordinator.

Across management scenarios (i-iv), carrying capacity was set to 200 individuals. This is the target population size as specified in the sihek SSP and reflects an optimistic scenario of expanding current capacity at breeding facilities. This high carrying capacity allows us to fully explore the effects of the different breeding management scenarios through modelling the different reproductive rates, with carrying capacity then only restricting *λ_s_* in model scenarios with the highest reproductive rates.

**Supplementary Appendix 4. Population viability analysis - model input parameter values**

**Table S4.** Parameter values for the average management model (i.e. baseline model), implemented in Vortex v10.2.17.0 ^12^.

| **Parameter** | **Estimate** | | | |
| --- | --- | --- | --- | --- |
| **Model structure** | | | | |
| Starting population size | 136 | | | |
| Carrying capacity | 200 | | | |
| Starting sex-age distribution | Female | | Male | |
| Age 1 | 8 | | 4 | |
| Age 2 | 14 | | 10 | |
| Age 3 | 5 | | 3 | |
| Age 4 | 4 | | 3 | |
| Age 5 | 4 | | 6 | |
| Age 6 | 2 | | 8 | |
| Age 7 | 5 | | 13 | |
| Age 8 | 2 | | 4 | |
| Age 9 | 3 | | 6 | |
| Age 10 | 3 | | 6 | |
| Age 11 | 3 | | 1 | |
| Age 12 | 2 | | 3 | |
| Age 13 | 2 | | 1 | |
| Age 14 | 0 | | 6 | |
| Age 15 | 0 | | 3 | |
| Age 16 | 0 | | 0 | |
| Age 17 | 0 | | 0 | |
| Age 18 | 0 | | 0 | |
| Age 19 | 0 | | 1 | |
| Age 20 | 0 | | 1 | |
| **Demography** | | | | |
| Reproductive system | long-term monogamy | | | |
| Pair duration | 3 years | | | |
|  | female | | male | |
| Age of first offspring | 3 | | 4 | |
| Age of last offspring | 12 | | 19 | |
| Max. lifespan | 23 | | | |
| Max broods per year | 5 | | | |
| Max progeny per brood | 3 | | | |
| Birth sex ratio | 0.5 | | | |
| % adult females producing 1+ hatchlings per year (±SD) | 35±14 | | | |
| Distribution of broods per year given production of 1+ hatchlings (%): | | | | |
| 1 brood | 47 | | | |
| 2 broods | 30 | | | |
| 3 broods | 16 | | | |
| 4 broods | 6 | | | |
| 5 broods | 1 | | | |
| Distribution of hatchlings per female per brood (%): | | | | |
| 1 offspring | 65 | | | |
| 2 offspring | 34 | | | |
| 3 offspring | 1 | | | |
| % adult males breeding | 100 | | | |
| **Sex-age class survival probabilities:** | Females | | Males | |
|  | Age 0-1 (*ɸ_1_*) | 0.63 | Age 0-1 (*ɸ_1_*) | 0.63 |
|  | Age 1-2 (*ɸ_2_*) | 0.94 | Age 1-2 (*ɸ_2_*) | 0.91 |
|  | Age 2-3 (*ɸ_3_*) | 0.91 | Age 2-3 (*ɸ_3_*) | 0.93 |
|  | Age 3+ (*ɸ_F_*) | 0.79 | Age 3-4 (*ɸ_4_*) | 0.94 |
|  |  | | Age 4+ (*ɸ_M_*) | 0.84 |
| Correlation in environmental variation between reproduction and survival | 0 | | | |
| **Genetics** | | | | |
| Inbreeding load (*B*) on 1^st^ year survival (±SD) i.e. GS1 | 8.43 ±3.44 | | | |
| Inbreeding load (*B*) on adult female survival (±SD) i.e. GS2 | 2.82 ±0.94 | | | |
| Inbreeding load (*B*) on adult male survival (±SD) i.e. GS3 | 5.54 ±0.98 | | | |
| Initial population mean inbreeding coefficient (*f*) | 0.08 | | | |
| Pair according to mean kinships | Static mean kinship list | | | |
| Prevent matings with kinships | >0.25 | | | |

**Table S5.** Functions used in PVA models to apply inbreeding load *B* to mortality probabilities. *B* is applied to the expected outbred mortality probability given observed ɸ*_s_*, *B* and initial population mean inbreeding coefficient (*f*) (i.e. 0.08), following Morton, Crow, & Muller (1956). GS1-3 are our estimates of *B* ±SD (Table S1). I is the *f* of the simulated individual.

| **Parameter** | **function** |
| --- | --- |
| 1^st^ year mortality | =100-((EXP(LN(*ɸ_1_*)+(GS1*0.08)))*EXP(-GS1*I)*100) |
| Adult female mortality | =100-((EXP(LN(*ɸ_F_*)+(GS2*0.08)))*EXP(-GS2*I)*100) |
| Adult male mortality | =100-((EXP(LN(*ɸ_M_*)+(GS3*0.08)))*EXP(-GS3*I)*100) |

**References**

1. Haig, S. M., Ballou, J. D. & Casna, N. J. Genetic identification of kin in Micronesian kingfishers. *J. Hered.* **86,** 423–431 (1995).

2. Haig, S. M. & Ballou, J. D. Genetic diversity in two avian species formerly endemic to Guam. *Auk* **112,** 445–455 (1995).

3. Hedrick, P. W. & Garcia-Dorado, A. Understanding Inbreeding Depression, Purging, and Genetic Rescue. *Trends Ecol. Evol.* **31,** 940–952 (2016).

4. Ballou, J. D. & Lacy, R. C. in *Population management for survival and recovery* (eds. Ballou, J. D., Gilpin, M. & Foose, T. J.) 76–111 (Columbia University Press, 1995).

5. Jenkins, J. M. in *Ornithological Monographs 31* (The American Ornithologists’ Union, 1983).

6. Marshall, J. T. The endemic avifauna of Sapan, Tinian, Guam and Palau. *Condor* **51,** 200–221 (1949).

7. Zou, G. A Modified Poisson Regression Approach to Prospective Studies with Binary Data. *Am. J. Epidemiol.* **159,** 702–706 (2004).

8. Nakagawa, S., Johnson, P. C. D. & Schielzeth, H. The coefficient of determination R2 and intra-class correlation coefficient from generalized linear mixed-effects models revisited and expanded. *J. R. Soc. Interface* **14,** (2017).

9. Lüdecke, D. Package ‘performance’: Assessment of Regression Models Performance. **14,** (2020).

10. Signorell, D. *et al.* Package ‘ DescTools ’: Tools for Descriptive Statistics Version. (2020).

11. Lacy, R. C., Miller, P. S. & Traylor-Holzer, K. Vortex 10 user’s manual. (2017).

12. Lacy, R. C. & Pollak, J. P. Vortex: A stochastic simulation of the extinction process. (2017).

13. Morton, N. E., Crow, J. F. & Muller, H. J. An estimate of the mutational damage in man from data on consanguineous marriages. *Proc. Natl. Acad. Sci. USA* **42,** 855–863 (1956).
